# The Determination of the Rapid and Effective Activity of an Air Sanitizer against Aerosolized Bacteria Using a Room-Sized Aerobiology Chamber

**DOI:** 10.3390/microorganisms12102072

**Published:** 2024-10-16

**Authors:** Bahram Zargar, M. Khalid Ijaz, Anthony Kevek, Mark Miller, Julie McKinney, Syed A. Sattar

**Affiliations:** 1 CREM Co. Labs, Units 1–2, Mississauga, ON L4V 1T4, Canada; bzargar@cremco.ca; 2 Global Research and Development for Lysol and Dettol, Reckitt Benckiser LLC, Montvale, NJ 07645, USA; 3 Faculty of Medicine, University of Ottawa, Ottawa, ON K1H 8M5, Canada

**Keywords:** bactericidal activity, dipropylene glycol, indoor air sanitization, *Klebsiella pneumoniae*, room-sized aerobiology chamber, *Staphylococcus aureus*

## Abstract

Air sanitization is an important non-pharmaceutical intervention for mitigating the risk of indoor pathogen spreading. A dipropylene glycol-containing air sanitizer was tested against aerosolized *Staphylococcus aureus* and *Klebsiella pneumoniae*. The bacteria, suspended in a soil load, were aerosolized using a six-jet Collison nebulizer with pressurized air. The 25-m^3^ (~900 ft^3^) aerobiology chamber was maintained at 22 ± 2 °C and 50 ± 5% relative humidity per the U.S. Environmental Protection Agency’s 2012 Guidelines on air sanitizers. An initial 2-min air sample was collected from the chamber using a slit-to-agar sampler containing 150-mm Petri plates, with Trypticase soy agar (TSA) containing neutralizers to quench the microbicidal activity of the air sanitizer, to determine the initial bacterial challenge in the air. The air sanitizer was sprayed into the chamber from pressurized cans. Additional air samples were collected from the chamber over 10 min to detect surviving bacteria. The TSA plates were then incubated aerobically at 36 ± 1 °C for 90 ± 4 h and scored for bacterial colony-forming units. A 30-s spray of the air sanitizer reduced infectious *S. aureus* and *K. pneumoniae* titers by 3.0 log_10_ (99.9%) in 3.2 ± 0.3 min and 1.2 ± 0.0 min, respectively. Based on these findings, the EPA granted registration of the air sanitizer as the first product of its kind for indoor air sanitization.

## 1. Introduction

The importance of non-pharmaceutical interventions intended to limit the spread of airborne infectious agents indoors has been noted during the recent COVID-19/SARS-CoV-2 pandemic [1]. These interventions have included, in various geographical regions and at various times during the pandemic, the wearing of masks, contact tracing, social distancing and avoidance of crowded indoor spaces, business and school closures, the heightened adoption of hand and surface hygienic measures, and improving indoor air ventilation [1]. In fact, a variety of pathogens can survive in and be transmitted by indoor air [1,2,3,4]. The risk of such transmission increases in settings with poor ventilation and under crowded conditions. While improved ventilation may reduce such risks, additional options include the use of HEPA filtration and face masks [5] and the application of technologies for the sanitization of indoor air, such as ultraviolet (UV) irradiation [6,7] or the use of formulated microbicidal sprays [6,8,9]. 

The latter approach is not new. However, advancements in the area have awaited the availability of safe air sanitization technologies as well as the development of suitable room-sized aerobiology chambers to perform realistic antimicrobial activity testing. To address these issues, we first formulated a non-toxic spray formulation and then tested it against airborne bacteria, as described here. 

The aerobiology test protocol described here complies with the 2012 Guidelines [10] of the EPA, and it has also been implemented within the standards of the ASTM International [11] and of the Association of Home Appliance Manufacturers (AHAM) [12].

## 2. Materials and Methods

### 2.1. Challenge Bacteria, Nebulization, and Bacterial Detection System

*Staphylococcus aureus* (ATCC # 6538) and *Klebsiella pneumoniae* (ATCC # 4352) were obtained from Cedarlane Labs (Burlington, ON, Canada), the Canadian agents for the American Type Culture Collection (ATCC, Manassas, VA, USA). Trypticase soy broth (TSB, MilliporeSigma, Oakville, ON, Canada) and Trypticase soy agar (TSA, MilliporeSigma) were used as the growth media for the bacteria. Working cultures were prepared by inoculating bacteria into 10 mL of TSB and incubating at 36 ± 1 °C for 18 ± 2 h. The cultures were adjusted by dilution in phosphate-buffered saline (PBS, MilliporeSigma) to deliver 4.2 to 5.0 log_10_ CFU/m^3^ during nebulization. The bacteria were prepared for aerosolization in a six-jet Collison nebulizer as PBS solutions containing the bacteria suspended within a tripartite soil load. The latter, often referred to as organic load, is intended to mimic human pathophysiological body fluids (enteric or respiratory) such as mucus, saliva, and fecal matter [13,14]. The nebulization solutions consisted of PBS (pH 7.2 ± 0.2) containing the soil load (bovine serum albumin [BioShop Canada, Burlington, ON, Canada] at 3.7 mg/mL final concentration, yeast extract [Fisher Scientific, Ottawa, ON, Canada] at 5.2 mg/mL final concentration, and bovine mucin [MP Biomedicals, Solon, OH, USA] at 12 mg/mL final concentration), 10 μL of Antifoam A (MilliporeSigma) to prevent foaming, and 50 μL of bacteria sufficient to achieve 4.2 to 5.0 log_10_ CFU/m^3^ in a total nebulization volume of 15.00 mL. 

The agar medium used in the programmable slit-to-agar (STA) air sampler (Particle Measuring Systems, Boulder, CO, USA) consisted of TSA plates supplemented with 0.07% lecithin (MilliporeSigma), 0.5% Polysorbate 80 (MilliporeSigma), and 0.2% sodium thiosulfate (MilliporeSigma) as neutralizers (used to quench the microbicidal activity as air was sampled from the aerobiology chamber directly onto the TSA plates). The STA samplers collected air samples from the middle of the aerobiology chamber in real time at the rate of 28.3 L (1 ft^3^)/min. The exposed plates were incubated aerobically at 36 ± 1 °C and scored for colony-forming units (CFU) after 18 ± 2 h and incubated again for an additional three days to allow for the development of slower-growing stressed or injured bacterial cells [15]. The culture media used in this study were tested for their ability to support the growth of *S. aureus* and *K. pneumoniae* by the inoculation of a plate or tube with ≤100 CFU of the test microorganism, followed by incubation at 36 ± 1 °C for 18 ± 2 h. The presence of microbial growth indicated the suitability of the culture media. Colony morphology and viability were confirmed at ≤100 CFU using the prepared nebulization fluid for all test dates on the culture media.

### 2.2. Evaluation of Neutralization Effectiveness of Sampling Medium

The approach taken to demonstrate that the neutralizing agents (lecithin, Polysorbate 80, and sodium thiosulfate) included in the air sampling agar media could adequately quench the bactericidal activity of the air sanitizer during air sample collection by the STA samplerhas been described in the Supplementary Materials section of this paper.

The air sanitizer evaluated was Lysol Air Sanitizer (Reckitt, Montvale, NJ, USA), a commercially available ready-to-use indoor air sanitizing spray presented in pressurized cans and containing 13% dipropylene glycol (DPG) tested at the lower certified limit [16]. At least three cans from each of the three separate batches (designated 1, 2, and 3) were provided to the testing facility (CREM Co. Labs, Mississauga, ON, Canada). At the testing facility, the air sanitizer containers were stored under ambient conditions until used in the aerobiology experiments. A glovebox on one side of the aerobiology chamber allowed access to the inside of the chamber without breaching the containment barrier and enabled operators to administer the air sanitizer into the chamber. The outlet of the air sanitizer spray container was pointed by the operator towards the chamber’s ceiling, and spraying was allowed to continue for 30 s while moving the container in a sweeping motion. The container was weighed immediately before and after each administration to determine the amount, in grams, of the air sanitizer discharged into the chamber.

### 2.3. Preliminary Studies

In a preliminary study, the concentrations of DPG in the aerobiology chamber air following a 30-s spray of the air sanitizer in the absence of bacterial challenge were determined by chamber air sampling at 5-min intervals. Resulting DPG concentrations were determined using a gas chromatography method [17]. A second preliminary study examined the relationship between the duration of air sanitizer spraying into the aerobiology chamber and the corresponding bactericidal efficacy against *S. aureus*. The air sanitizer was sprayed for 0–30 s in six independent experiments involving spraying of the air sanitizer in increments of 5 s (i.e., 5 s, 10 s, 15 s, 20 s, 25 s, and 30 s) into the chamber, which had previously been challenged with aerosolized *S. aureus*. Each spray duration was evaluated in a separate experiment, in which air samples were collected for 10 min following the spraying and assayed for surviving *S. aureus*, and the log_10_ reductions for each spray duration were calculated and plotted.

### 2.4. Experimental Design

Certain aspects of the experimental design, as dictated by the EPA-approved aerobiology protocol, were followed. These included the characteristics of the aerobiology chamber to be used (at least the size of a typical room, e.g., 10 ft × 10 ft × 8 ft), the requirement to monitor atmospheric conditions (temperature and relative humidity [RH]), and the specific challenge bacteria (see below) to be used. The design and characteristics of the aerobiology chamber used in this study have been described previously [3,18,19] and met the requirements set forth by the EPA.

In brief, the bacteria (*S. aureus* or *K. pneumoniae*) suspended in a soil load to simulate the presence of body fluids were nebulized into the aerobiology chamber for 10 min. After a 5 min mixing time to ensure the even distribution of the aerosolized bacteria, baseline bacterial concentrations were obtained by air sampling, and air sampling continued in order to evaluate the natural rate of decay of the bacteria within the aerosols. In separate tests, after the 5-min mixing time to ensure the even distribution of the aerosolized bacteria and the sampling of the baseline bacterial concentration, the test air sanitizer was sprayed into the chamber over a 30-s period. In each case, air sampling then occurred for varying time periods (in the control test, each sample was taken over 2 min, and in the microbicidal efficacy test, sampling occurred for 10 min post spraying).

Per the EPA Guidelines [10], “The test results should demonstrate a viable bacterial count reduction of ≥99.9 percent (≥3 log_10_ reduction) over the parallel untreated control, after correcting for settling rates, in the air of the test enclosure with each of the required bacteria”.

## 3. Results

### 3.1. DPG Decay Curve in Chamber Air Following Spraying Air Sanitizer into the Aerobiology Chamber

**Figure 1:**
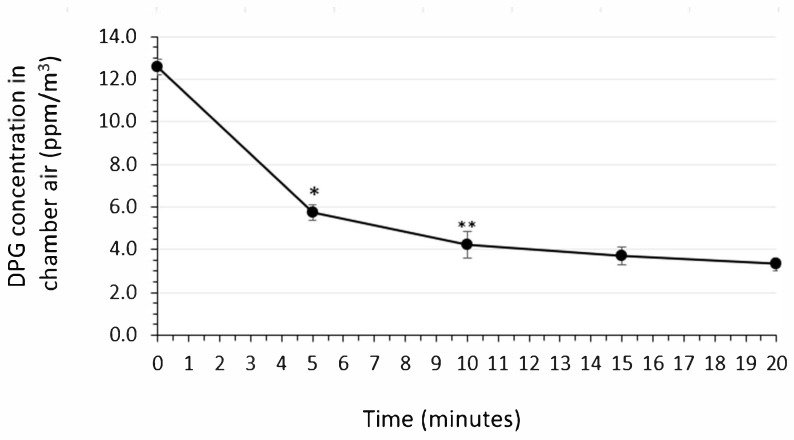
Decay curve of dipropylene glycol (DPG) in the chamber air following a 30-s spray of the air sanitizer. Values shown are the means ± standard deviations for *n* = 3 determinations per time point, with each point on the graph representing the efficacy outcome from an independent test run for that spray duration (i.e., 5 s, 10 s, 15 s, 20 s, 25 s, and 30 s). *, Significantly different from time 0 min value, *p* < 0.05; **, significantly different from time 5 min value, *p* < 0.05 (two-tailed *t*-test assuming equal variances).

Following a 30-s spraying of the air sanitizer into the aerobiology chamber, air samples were obtained at 5 min intervals and analyzed for DPG concentration for 20 min post spraying. The resulting DPG decay curve is shown in Figure 1. Immediately after spraying, the determined DPG concentration was 12.6 ppm, and the concentration decreased to 3.3 ppm at the end of 20 min.

### 3.2. Relationship between Air Sanitizer Spray Duration and Bactericidal Efficacy against S. aureus

**Figure 2:**
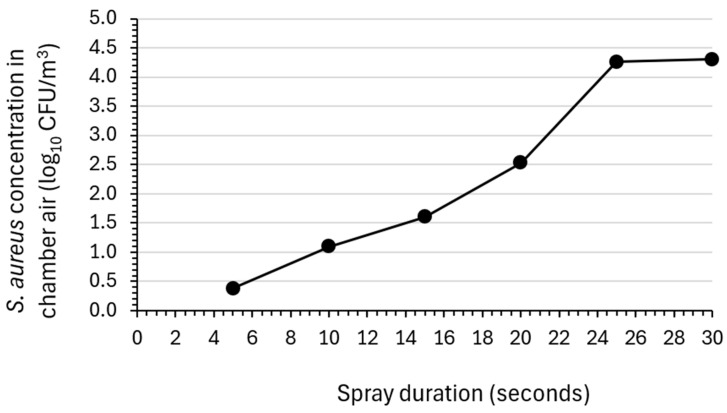
Dose response for the bactericidal efficacy of varying durations of spraying the air sanitizer against *S. aureus*. Values indicate the determined log_10_ reductions in *Staphylococcus aureus* (*S. aureus*) titer in the aerobiology chamber air samples collected 10 min following the indicated spray durations (*n* = 1 per spray duration).

Figure 2 shows the relationship between the air sanitizer spray duration in seconds and the resulting bactericidal activity against *S. aureus*. In six separate experiments, the chamber air was challenged with aerosolized *S. aureus*, after which the air sanitizer was sprayed into the aerobiology chamber in increments of five seconds (i.e., 5 s, 10 s, 15 s, 20 s, 25 s, and 30 s). Samples of air from the chamber were collected for ten minutes following the spray duration being evaluated and were assayed for residual *S. aureus*. As shown in Figure 2, there was a direct correspondence between the duration of the spray and the determined bactericidal activity. 

### 3.3. Evaluation of Survival of the Aerosolized Bacteria in the Aerobiology Chamber in the Absence of the Air Sanitizer

**Figure 3:**
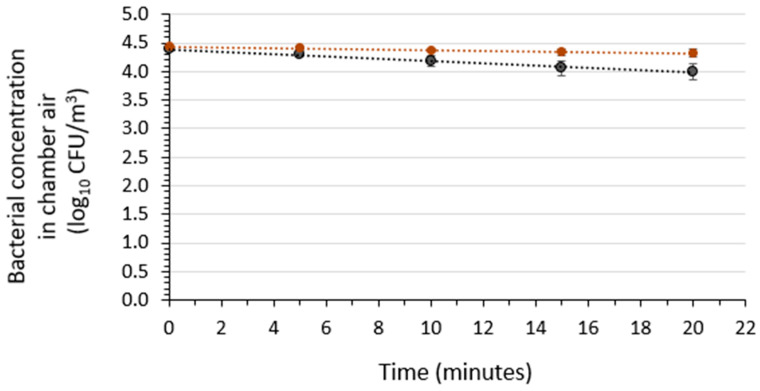
Bacterial concentration in the air of the aerobiology chamber following aerosolization from suspending medium containing the soil load in the absence of the air sanitizer. Three independent runs were performed. A plot of the average results from the three runs for each type of bacteria (black symbols, *Klebsiella pneumoniae*; red symbols, *Staphylococcus aureus*) is included, along with the standard deviations. The associated linear regression lines for the control average plots are shown in the figure, and the equations are *y* = −0.0201*x* + 4.3910 (R^2^ = 0.9917) and *y* = −0.0053*x* + 4.4327 (R^2^ = 0.9774) for *K. pneumoniae* and *S. aureus*, respectively.

The viability of the test bacteria in the air of the chamber over a 20-min period was determined in three independent runs for each of the two bacteria species (Figure 3), performed at 20 °C to 25 °C and 50 ± 5% RH. The results indicate log-linear (first-order) viable aerosolized bacterial concentration reductions over time. The average decay rates for *S. aureus* and *K. pneumoniae* in the presence of the soil load were determined to be 0.0053 ± 0.0027 log_10_ CFU/m^3^/min and 0.0201 ± 0.0066 log_10_ CFU/m^3^/min, respectively. Over the 10-min duration of the efficacy studies to be performed in the presence of the soil load, this indicated that less than a 0.2-log_10_ reduction in viable bacterial concentration was to be expected in the absence of the air sanitizer. 

### 3.4. Neutralization Effectiveness Testing

The neutralization effectiveness testing (Supplementary Materials) demonstrated that the neutralizing ingredients (lecithin, Polysorbate 80, and sodium thiosulfate) contained in the air sampling medium adequately quenched the bactericidal activity of the tested air sanitizer. This enabled the determination of the time kinetics of aerosolized viable bacterial concentration reduction following the application of the test air sanitizer in the aerobiology chamber. 

### 3.5. Assessment of the Air Sanitizer’s Activity against Klebsiella pneumoniae

**Figure 4:**
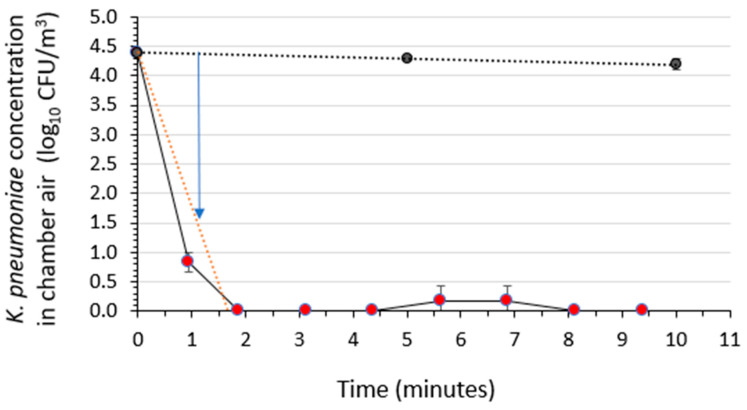
Time kinetics for the bactericidal activity of the air sanitizer against aerosolized *Klebsiella pneumoniae* suspended in a medium containing a soil load in a room-sized aerobiology chamber. The black symbols indicate mean ± standard deviation (*n* = 3 independent runs) for the stability of aerosolized *K. pneumoniae* in the absence of the air sanitizer. The red symbols represent the bacterial concentration vs. time during efficacy testing of three sanitizer batches (mean ± standard deviation for *n* = 9 independent runs). The blue arrow shows the time associated with a 3-log_10_ reduction in viable concentration, accounting for the decay of the bacteria in the absence of the air sanitizer. The black dotted line displays the linear regression line fitting the *K. pneumoniae* concentration in the absence of the air sanitizer. The red dotted line shows the linear regression line fitting the *K. pneumoniae* concentration in the presence of the air sanitizer.

The time kinetics of bactericidal activity (reduction in viable concentration) of *K. pneumoniae* in a room-sized aerobiology chamber’s air over a 10-min period following a 30-s spraying of the test air sanitizer was determined in three independent runs for each of the three sanitizer batches. This testing was performed with the chamber’s air maintained at 22 ± 2 °C and 50 ± 5% RH. The results, based on a mean of nine efficacy tests and three control tests, are displayed in Figure 4 (three independent runs for each of the three sanitizer batches). In each experiment, the log_10_ CFU/m^3^ was calculated at each sampling time. The mean and standard deviation of the nine efficacy tests and three control tests were calculated at each sampling time. The average activity (air sanitizer) curve and average control concentrations were fitted with a linear regression line, and the time required for achieving a 3-log_10_ reduction in viable *K. pneumoniae* concentration was determined by subtracting the average activity curve from the average control curve. A 3.0-log_10_ (99.9%) reduction in the viable concentration of aerosolized *K. pneumoniae* was achieved within 1.2 ± 0.0 min of exposure to the air sanitizer.

### 3.6. Assessment of the Air Sanitizer’s Activity against Aerosolized Staphylococcus aureus

**Figure 5:**
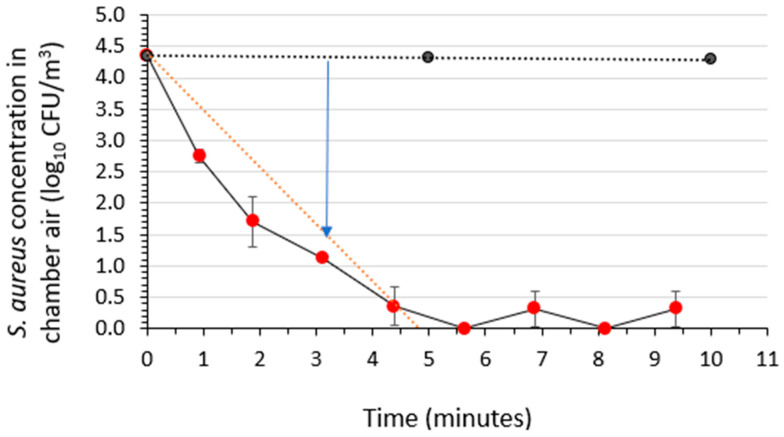
Time kinetics for bactericidal activity of the air sanitizer against aerosolized *Staphylococcus aureus* suspended in a medium containing a soil load in a room-sized aerobiology chamber. The black symbols indicate mean ± standard deviation (*n* = 3 independent runs) for the stability of aerosolized *S. aureus* in the absence of the air sanitizer. The red symbols represent the bacterial concentration vs. time during efficacy testing of the three sanitizer batches (mean ± standard deviation for *n* = 9 independent runs). The blue arrow shows the time associated with a 3-log_10_ reduction in viable concentration, accounting for the decay of the bacteria in the absence of the air sanitizer. The black dotted line displays the linear regression line fit to the *S. aureus* concentration in the absence of the air sanitizer. The red dotted line shows the linear regression line fitting the *S. aureus* concentration in the presence of the air sanitizer.

The time kinetics of bactericidal activity (reduction in viable concentration) of *S. aureus* in a room-sized aerobiology chamber’s air over a 10-min period following a 30-s spraying of the test air sanitizer was determined in three independent runs for each of the three sanitizer batches. This efficacy testing was performed at 22 ± 2 °C and 50 ± 5% RH. The average results for the air sanitizer batches are displayed in Figure 5. 

The plot shows the average (*n* = 9) log_10_ concentration vs. time post spraying along with the standard deviations. The average activity (air sanitizer) curve and average control curve were fit with a linear regression line, and the time required for achieving a 3-log_10_ reduction in viable *S. aureus* concentration was determined by subtracting the average activity curve from the average control curve. A 3-log_10_ (99.9%) reduction in viable aerosolized *S. aureus* concentration was achieved within 3.2 ± 0.3 min of spraying the air sanitizer.

## 4. Discussion

One might expect the level of viable pathogenic bacteria in indoor air in a given setting to vary from time to time, depending on a variety of factors, including the relative crowding of the spaces, the presence of infected individuals and the stage of the infection, and the adequacy of air changes, etc. [20,21,22,23]. Empirical data available from published surveys suggest that the concentrations of viable aerobic bacteria in indoor air in domestic settings may range between 10 and 5036 CFU/m^3^ [4,23]. In view of this, the product performance criterion for this study followed the ≥3-log_10_ reduction required by the EPA in the level of viability of the airborne bacteria experimentally introduced into a room-sized aerobiology chamber. The air sanitizer evaluated achieved a 3-log_10_ reduction (99.9%) of *S. aureus* and *K. pneumoniae* within 3.2 ± 0.3 min and 1.2 ± 0.0 min, respectively, and achieved 4-log_10_ reductions (99.99%) within 4.2 ± 0.4 min and 1.5 ± 0.0 min, respectively. 

*Staphylococcus aureus* was selected as a challenge bacterium because it is widely accepted as a surrogate for Gram-positive cocci in aerobiology tests. The bacterium is relatively stable in indoor air and is readily transmitted via air [24]. *Klebsiella pneumoniae* was chosen as a challenge organism because regulatory agencies (e.g., the EPA) mandate its use as a surrogate for Gram-negative bacilli in assessing indoor air decontamination. 

The Collison nebulizer used in this study has been well-recognized for its ability to generate aerosols in the respirable size range [25]. It produces droplets with a mass median aerodynamic diameter (MMAD) of 1.58 µm with a geometric standard deviation (GSD) of 1.85 and, thus, better reflects the health risks associated with the direct inhalation of airborne pathogens [26]. The STA sampler provides direct and event-related CFU counts in the recovery plates and has been found to be more effective than liquid impactors [27].

As far as we are aware, this DPG-based air sanitizer is the first air sanitizer to have been registered with the EPA for indoor air sanitization [28]. In their announcement [28], the U.S. EPA stated that the “EPA conducted a robust risk assessment on exposure from both household and commercial use. When used following label directions, this product poses no unreasonable adverse risks to human health or the environment”. In addition, safety studies have been conducted on the spray formulation investigated here. It has been concluded that the formulation presents no concerns from an acute oral or inhalation point of view. The formulation is classified as a mild irritant and is considered non-sensitizing (Reckitt, unpublished data). Based on the available toxicological and safety data for the ingredients, the spray formulation is considered suitable for the intended and foreseeable conditions of use when applied in accordance with the product label. Dipropylene glycol and other types of glycols have been used in the manufacture of a wide variety of drugs, cosmetics, and foods, based on their generally low human and environmental toxicities [28,29,30,31]. Despite this, as a precaution, the air sanitizer evaluated in this study is intended for use in unoccupied spaces to avoid human exposure by inhalation. 

The mechanism of bactericidal action in DPG and related glycols remains to be explored fully. However, recent ultrastructural studies [32] indicate that DPG-containing sprays produce pronounced cellular disruption of both Gram-positive (e.g., *S. aureus*) and Gram-negative (e.g., *K. pneumoniae*) bacterial cells, with the release of cytoplasmic components. It should be noted that DPG and other types of glycols show virtually no microbicidal activity in liquid form, allowing for the widespread use of this ingredient in liquid form in medicines, cosmetics, and foods [29,30,31]. In contrast, as shown here, the formulated DPG-based air sanitizer, when sprayed, exerts a rapid and pronounced bactericidal effect against aerosolized bacteria in an aerobiology chamber. In separate studies [33], we have demonstrated that this DPG-containing air sanitizer also displays virucidal activity against aerosolized coliphage MS2 (used as a surrogate for small, non-enveloped respiratory and enteric viruses such as adenoviruses, rhinoviruses, and noroviruses) and cystovirus Phi6 (used as a surrogate for enveloped viruses such as influenza virus, respiratory syncytial virus, and human coronaviruses, including SARS-CoV-2). 

## 5. Conclusions

In summary, a collaborative effort between the authors led to the development and approval of a protocol by the EPA for assessing the bactericidal efficacy of a DPG-based air sanitizer and, subsequently, enabled the registration of the product by the EPA. The protocol is now an ASTM International standard, and a method approved by the AHAM that may be used, along with similar experimental set-ups and aerobiology chambers, to assess indoor air decontamination technologies based on technologies such as UV irradiation, chemical fogging, and HEPA filtration. The protocol has also been successfully adapted for other types of airborne human pathogens, such as viruses, fungi, and mycobacteria.

## Data Availability

Data are contained within the article and Supplementary Materials.

## Supplementary Materials

The following supporting information can be downloaded at https://www.mdpi.com/article/10.3390/microorganisms12102072/s1, Approach for evaluating neutralization effectiveness; Results of neutralization effectiveness testing; Table S1: Neutralization effectiveness testing of air sample medium: *S. aureus*; Table S2: Neutralization effectiveness testing of air sample medium: *K. pneumoniae*.
