# Supplementary material for: The Determination of the Rapid and Effective Activity of an Air Sanitizer against Aerosolized Bacteria Using a Room-Sized Aerobiology Chamber
Source: Microorganisms. 2024 Oct 16;12(10):2072. doi: 10.3390/microorganisms12102072 (PMC11510681; doi:10.3390/microorganisms12102072)
Supplement: Supplementary file 1 [file microorganisms-12-02072-s001.zip › microorganisms-3205732-supplementary.pdf]

Supplementary Materials

Determination of Rapid and Effective Activity of an Air Sanitizer against Aerosolized Bacteria using a Room-sized Aerobiology Chamber

Approach for evaluating neutralization effectiveness

In order to enable the determination of the time-kinetics of aerosolized viable bacterial concentration reduction following application of the test air sanitizer, it was necessary to demonstrate that the neutralizing agents included in the air sampling agar medium (lecithin, Polysorbate 80, and sodium thiosulfate) could adequately quench the bactericidal activity of the air sanitizer. The approach taken was to spray the air sanitizer into the chamber for 45 seconds and then to take an air sample after 10 minutes (the longest air sampling time planned for this study) using the STA sampler containing a TSA agar plate supplemented with neutralizers. The recovery plate was then inoculated with 100 µL of the test bacterial suspension (*Staphylococcus aureus* or *Klebsiella pneumoniae*) diluted to yield a countable number of colonies. For controls, one sample plate unexposed to the air sanitizer was similarly inoculated with the diluted bacteria. A sample plate not exposed to bacterial aerosol was used as a sterility control. The plates were incubated at 36 ± 1°C and after 18±2 hours and at the end of an additional three days were scored for colony-forming units (CFU). The neutralizing ingredients were regarded as effective if the numbers of CFU on the plates exposed to air sanitizer were not more than 50% different from the CFU on the control plate.

Results of neutralization effectiveness testing

In the case of *S. aureus*, the average number of recovered colonies for bacteria exposed to the air sanitizer was within 15% of the average number for the control plates. For *K. pneumoniae*, the average number of recovered colonies for bacteria exposed to the air sanitizer was within 9% of the average number for the control plates. In either case, it was concluded that the neutralizing ingredients contained in the sampling medium adequately quenched the bactericidal activity of the test air sanitizer.

Table S1: Neutralization effectiveness testing of air sample medium: *S. aureus*

Sample ID	Control (Non-exposed)		Neutralizer (Exposed)
Replicate No.	1	2	1
No. of CFU	60, 41, 44	61, 37, 43	46, 31, 46
Average	48		41
% Difference	15%		

Table S2: Neutralization effectiveness testing of air sample medium: *K. pneumoniae*

Sample ID	Control (Non-exposed)		Neutralizer (Exposed)
Replicate No.	1	2	1
No. of CFU	66, 75, 59	64, 86, 65	59, 69, 62
Average	69		63
% Difference	9%		
